# Dr. Ephraim Ingals

**Published:** 1901-01

**Authors:** 


DR. EPHRAIM INGALS, one of the most prominent men in the early history of Chicago, Ill., died in that city December 18, 1900. He was born in Abington, Conn., on May 26, 1823. Dr. Ingals went to Chicago when a boy. He arose to prominence in his profession and as a citizen. He was a personal friend of Abraham Lincoln.

